# Dangerous Medication

**Published:** 1869-01

**Authors:** 


### DANGEROUS MEDICATION.

Not long since, a man who had borne a high reputation for honesty and propriety of deportment; who was implicitly trusted by the entire community in which he had lived for fifty years, committed one of the most brutal murders ever perpetrated in the town; it was the murder of a long-tried bosom friend. Men do not become utterly depraved in a moment, or a month, in the ordinary circumstances of a village life. The jury in the case seemed to think that the terrible deed must have been committed in a sudden burst of phrenzy or madness, and adjudged him not guilty of murder in the first degree, but of a less aggravated type of crime, and a long imprisonment was imposed. It was disclosed on the trial that the man had been in the habit of taking ether!

Within a few months, the community was shocked at the sudden death of a gentleman high in office and in the esteem of multitudes throughout the nation. On a legal investigation it was proved, that he had killed himself by inadvertently taking an overdose of chloroform which he had been in the habit of using on his own responsibility, when suffering from attacks of neuralgia, or from depression of spirits; at other times he would use it when he had pressing business to transact, and it was necessary to be wide awake.

A few days ago, one of the most honored men of a large city, who had once been its mayor, and who had for many years occupied various offices of trust and responsibility, was found dead in his bed in the morning, having retired in his usual good health. He had been accustomed to take laudanum, to promote sleep and for other purposes, and on this occasion had taken an over-dose.

Thousands of deaths occur every year from the unadvised use of dangerous medicines; it often arises in this way: a person is suffering, the family physician is called, he writes a prescription, it is taken, and instant and grateful relief is experienced; the patient desires to know the name of the marvellous remedy, bears it in mind, and if there is a recurrence of his ailment, or if there is something similar, he ventures to send (for the remedy) direct to the druggist; on being relieved again, he becomes enthusiastic and volunteers advice to his friends; they are relieved—sometimes! and forthwith he begins to think he knows "about as much as any of the doctors;" a little later, it is not unusual to see a record in the newspapers that Mr. ——— was "found dead in his bed this morning."

In the cases above, all were men of intelligence and position, and yet they allowed themselves to fall into the habitual use of the most dangerous remedies known to science. We would advise every one

1. Never to keep dangerous medicines in the house.
2. Never to use a dangerous drug, except by the immediate advice of your family physician.
3. When in the use of any remedy, you find yourself inclined to employ it oftener or in larger quantities to produce the same effects, whether it be spirits, tobacco, snuff, tea, coffee, chloroform, ether or any other stimulant, or poison, be assured that you are on the very verge of destruction, and that you are liable any day to an instant death.

4. When you find yourself inclined to "take" anything, even a cup of tea, or coffee, to enable you to perform any work in hand, mental or bodily, avoid it as you would a deadly poison. The three greatest names of this century in our country, in pulpit, bar and forum, died drunkards; and long before their deaths it was known to their friends, that they were "incapable of an effort" without being first "fortified" with a glass of brandy.

